# The Effect of Scaling and Root Planning on Salivary TNF-α and IL-1α Concentrations in Patients with Chronic Periodontitis

**DOI:** 10.2174/1874210601711010573

**Published:** 2017-10-31

**Authors:** Masoome Eivazi, Negar Falahi, Nastaran Eivazi, Mohammad Ali Eivazi, Asad Vaisi Raygani, Fatemeh Rezaei

**Affiliations:** 1 Department of Periodontics, School of Dentistry, Kermanshah University of Medical Sciences, , Iran; 2 School of Dentistry, Kermanshah University of Medical Sciences, , Iran; 3 Department of ENT, School of Medicine, Kermanshah University of Medical Sciences, , Iran; 4 Department of Pharmacoeconomics and Pharmaceutical Management, School of Pharmacy, Shahid Beheshti University of Medical Sciences,, Iran; 5 Department of Biochemistry, School of Medicine, Kermanshah University of Medical Sciences, , Iran; 6 Department of Oral Medicine, School of Dentistry, Kermanshah University of Medical Sciences, , Iran

**Keywords:** Chronic periodontitis, Non-surgical treatment, Saliva, TNF-α, IL-1α, ELISA method

## Abstract

**Objective:**

Periodontitis is one of the main diseases in the oral cavity that causes tooth loss. The host immune response and inflammatory factors have important role in periodontal tissue. The current study was done with the objective to determine the effect of scaling and root planning on the salivary concentrations of tumor necrosis factor-alpha (TNF-α) and interleukin-1-alpha (IL-1α).

**Methods:**

In this quasi-experimental clinical trial, 29 patients with chronic periodontitis and 29 healthy subjects without periodontitis were studied. Clinical examination findings and salivary TNF-α and IL-1α (using ELISA method) were compared before and after scaling, root planning.

**Results:**

Before starting treatment, salivary TNF-α and IL-1α concentrations were higher in healthy control group than in periodontitis group (*P*< 0.05). Non-surgical treatment increased the concentration of these two biomarkers in the saliva. However, increase in IL-1α concentration was not statistically significant (*P*= 0.056). There was a negative relationship between TNF-α and IL-1α levels with pocket depth and attachment loss (*P*< 0.05).

**Conclusion:**

Scaling and root planning improved periodontal disease indices and salivary TNF-α and IL-1α levels.

## 1 INTRODUCTION

Periodontitis is one of the leading causes of tooth loss which usually occurs in those older than 40 years [1] . Microbial agents, environmental factors, and genetic causes play role in the development and progression of periodontitis [2]. Evidence shows that the prevalence of periodontitis varies globally. In Asian countries, this condition has been reported to be more prevalent and more severe [3]. Periodontitis affects about 50% of adult population in the US who age more than 30 years. The prevalence of periodontitis increases with aging [4]. Periodontitis is more common in males and also it is more severe in cigarette smokers [5, 6]. Periodontitis is not just a common oral disease. It reflects persistent immune system activation and is associated with conditions such as vascular diseases, and increased risk of cardiovascular diseases, diabetes, rheumatoid arthritis, and premature delivery [7-11]. Periodontitis risk factors include pathogens, lifestyle, psycho-social factors, chronic diseases, and genetic factors [12]. It is now widely accepted that main pathogenesis behind periodontitis is a polymicrobial process [13]. Gingival epithelial cells secrete inflammatory cytokines such as tumor necrosis factor-alpha (TNF-α), interleukin-1-alpha (IL-1α), IL-1β, TNF-β, IL-8, and IL-6 in response to pathogens [14].

Even though immune system activity to oppose pathogen factors is necessary, irregular immune system activation can result in chronic inflammation and consequently cause gradual destruction of periodontal tissues [15]. Imbalanced immune system response can be elicited due to genetic and epidemiologic factors such as gender, age, cigarette smoking, and systemic diseases [16, 17]. Cytokines are polypeptides that are produced in response to microbes and other antigens and modulate immune and inflammatory reactions. The effect of cytokines can be local or systemic. A cytokine can affect production and action of other cytokines [18]. IL-1 family has major role in initiation and continuation of immune and inflammatory responses [19-21]. IL-1 is a polypeptide with a wide range of actions such as modifying other inflammatory mediators including cytokines and prostaglandin E2, adhesion molecule expression regulation in endothelial cells, induction of osteoclast formation and stimulation inside bone marrow, induction of metalloproteinase release from macrophages and other cells [22]. IL-1 has two active forms: IL-1α and IL-1β. In addition to monocytes, other cells like macrophages, T lymphocytes, fibroblasts, vascular, skin, and brain cells can produce Il-1. IL-1α is also called pro-inflammatory factor as this activates genes related to immune and inflammatory systems.

TNF-α as a pro-inflammatory cytokine and immune system modulator has a major role in pathogenesis of various inflammatory conditions inside the human body [23, 24]. This inflammatory marker has some other minor activities such as inflammatory cells recruitment, bone resorption *via *IL-1, stimulation of granulocyte macrophage colony stimulating factor, collagen production inhibition, collagenase induction inside tissues, and induction of osteoclast differentiation *via *granulocyte macrophage colony stimulating factor [25-28]. Similarities between IL-1 and TNF effects are amazing as these two cytokines and their receptors are different structurally. TNF-α causes, directly or indirectly, bone resorption and its formation inhibition [29].

Non-surgical therapy including scaling and root planning is effective treatment for periodontal diseases [30]. Low intensity ultrasonic waves activate cellular growth signaling pathway and stimulate circulating angiogenic cells [31, 32].

Saliva is a complex fluid, produced by major and minor salivary glands. It contains composition such as has inflammatory mediators that play important role in oral health. Saliva composition, naturally or under certain conditions, varies in different individuals [33, 34]. The relationship between some inflammatory mediators in saliva and periodontitis pathogenesis has been established in some studies. The question rises here is that whether these biomarkers (TNF-α and IL-1α) can have relationship with success rate of non-surgical treatment for chronic periodontitis.

## 2 MATERIALS AND METHODS

A total of 58 subjects who presented to the Clinic of Dentistry School in Kermanshah (Iran) in 2015-16 were recruited for this quasi-experimental clinical trial. 29 subjects had chronic periodontitis based on definitions made by the American Academy of Periodontology and 29 subjects did not have periodontitis and gingivitis as a control group. All subjects were examined by a dentist (researcher). Inclusion criteria consisted of having at least 20 teeth, minimum age of 18 years and without any systemic diseases that can affect the periodontium. Inclusion criteria for periodontitis group also required presence of attachment loss and bone destruction in more than 30% of dental areas for at least 3 months [35]. Those who had received antibiotics in the last 2 months, long-term treatment by non-steroidal anti inflammatory drugs (NSAIDs), smokers, alcohol drinkers, periodontal treatments in the last 6 months, mucosal inflammatory conditions such as lichen planus or recurrent aphtus stomatitis, and pregnant or breastfeeding women were excluded.

The study protocol was approved by the Ethics Committee of Kermanshah University of Medical sciences, Kermanshah, Iran (KUMS REC 1395.128). The study protocol was registered at the Iranian Registry of Clinical Trials (IRCT2016052825649N3).

Firstly, the patients were examined and if inclusion criteria were met, they were informed orally about the study objectives. If agreed to participate, written informed consent was obtained from the patient. Periodontal indices such as pocket depth, attachment loss, bleeding on probing, and the plaque index were documented by a dentist before and 30 days after scaling. The pocket depth (the distance between gingival sulcus base and its margin) was measured in four locations using the Williams probe. Attachment loss, which is the distance between cementoenamel junction and gingival sulcus base, was determined by standard technique. The O’Leary plaque index was also documented.

Before non-surgical therapy, 5 mL of non-stimulated saliva was collected in sterile tubes using the method that Navazesh described in 2014 [36]. Saliva was collected when the patient was seated and head was slightly tilted forward with eyes open. It should be mentioned that the subjects were instructed not to chew gums, brush teeth, eating and drinking for one hour prior to collecting saliva samples. Also, participants washed their mouth for 2 minutes with normal saline prior to collecting saliva samples. The samples were stored at ice packages before transfer to the laboratory which was done up to 2 hours after getting the samples. The samples were centrifuged for 20 minutes at 2000-3000 rpm and after transfer to microtubes with cap were kept at - 20°C till ELISA test was done.

After collecting the primary sample, the patients underwent scaling and root planning and hygiene education. The scaling and root planning were done by ultrasonic device (WOODPECKER^®^ made in china) by a dentist. The second salivary sample was collected one month after scaling by the same method described above. The periodontal indices were assessed once more. As a period of 1-2 weeks is usually required to allow the injuries occurred during scaling to be healed and epithelialized [37], periodontal examination and collection of salivary samples were done after 30 days to ensure periodontal condition has improved. Salivary TNF-α and IL-1α levels were measured by ELISA method using diagnostic kits manufactured by Hangzhou EASTBIOPHARM Co., Ltd. For measurement of the concentrations, ELISA kits for serum, blood, and saliva samples with sensitivity 1.25 ng/L for TNF-α and 0.54 pg/mL for IL-1α were used.

The subjects of the control group were selected considering they were compatible with regard to age and gender with the periodontitis group. The control group did not have periodontitis or any of the exclusion criteria. After getting written informed consent, non-stimulation salivary samples were collected in the control group with the same instructions described previously.

The analyses were done by the SPSS software for Windows (ver. 16.0). In order to determine the normal distribution of the data, the Shapiro-Wilk test was used. If the data had normal distribution, the independent sample t-test was used to compare the data between periodontitis and control groups. If the data did not have a normal distribution, the Mann-Whitney U test was used for this purpose. In order to compare the data before and after non-surgical therapy, the Wilcoxon test was applied. In order to determine the relationship between the variables, the Spearman’s correlation test was used. The significance level was set at 0.05.

## 3 RESULTS

There were 13 males and 16 females in each group. Mean (±SD) ages of periodontitis and control groups were respectively 38.59 (±12.46) and 31.28 (±8.49) years. Comparison of periodontal clinical parameters in periodontitis group showed improvement after non-surgical treatment. Periodontal pocket depths before and after treatment were 2.4 (0.56) and 1.7 (1.65 to 2.11), respectively. Mean clinical attachment loss before treatment was 2.4 (0.92) which reached to 2.3 (1.7 to 2.7) after the treatment. Bleeding on probing was 2.9 (0.92) before therapy and reached to 15 (5.3 to 25) after treatment. Periodontal clinical index showed significant improvement after treatment (*P*< 0.001); (Table **1**).

Mean (SD) salivary TNF-α levels in periodontitis before and after the therapy and control group were 51.0 (17.7), 58.0 (15.33) and 72.4 (28.4) respectively. Comparison of mean TNF-α level between periodontitis and control groups before and after the treatment showed significant differences (*P*< 0.05). Salivary TNF-α concentration increased after the treatment, the change was statistically significant (*P*=0-02) (Table **2**).

Mean (SD) salivary IL-1α levels in periodontitis groups before and after the treatment and control group were 54.7 (31.5), 65.7 (38.31) and 97.5(66.04). Comparison of mean TNF-α level between periodontitis and control groups before and after the treatment showed significant differences (*P*< 0.05); Although, salivary IL-1α concentration increased after the treatment, the change was not statistically significant (*p*=0.056) (Table **2**).

There was a negative relationship between TNF-α and IL-1α levels with pocket depth and attachment loss (Table **3**).

## 4 DISCUSSION

TNF-α and IL-1α are two important cytokines in immune and inflammatory responses of the body. Bacterial lipopolysaccharides stimulate cellular receptors. After the ligation, intracellular messenger is activated and results in TNF and IL gene expression and some other cytokines. This process is the start of host response to pathogenic factors. Although destructive effects of these factors in inflammatory conditions have been reported in some studies, the beneficial and modifying effects of these factors in defense system of the host should not be underestimated. Comparison of clinical parameters such as PD, CAL, and BOP before and after scaling and root planning showed that all these items improved significantly. The results showed that salivary TNF-α level was lower in periodontitis group compared to control group. Non-surgical therapy (scaling and root planning) and omission of pathogenic factors resulted in increase in salivary TNF-α level and improvement in clinical indices such as pocket depth, attachment loss, and bleeding on probing. The result of study by Geng [38] and Rai [39] showed that TNF-α can be a biomarker for periodontal disease. However, in these two cross-sectional studies, higher level of TNF-α was reported in disease group compared to healthy group. In Rai study, patients with different types of periodontitis were studied. Here, we only included patients with chronic periodontitis to have more reliable results and this group was compared to gender-matched healthy group as control group. Similar to our result, in Ulker [40], Ng [41], and Aurer [42] studies, patients with periodontal disease were reported to have lower level of TNF-α.

In Aurer study on different groups including chronic periodontitis patients, healthy group, and patients with tooth loss showed that in addition to TNF-α, salivary levels of other inflammatory factors such as C3, CRP, and α-2M were lower in periodontitis group compared to other groups. The reduced levels of inflammatory proteins show that in such chronic inflammatory diseases, host immune response has been decreased [43, 44].

In addition to the mentioned studies, Shy [30] study reported increase in TNF-α level after non-surgical treatment and improvement in clinical indices. Despite common belief that periodontal pathogens cause progressive destruction of periodontal tissue *via *severe immune response induction, some *in vitro* studies showed that periodontal pathogens, compared to non-pathogenic factors, cause lower level of inflammatory cytokines induction [45-50]. **In vitro** study by Dickinson showed that Porphyromonas gingivalis which is usually seen in severe and chronic periodontitis has high potential to invade several epithelial layers but does not stimulate pro-inflammatory cytokines production. Streptococcus gordonii, which is dominantly existent on epithelial surface, causes continuous secretion of cytokines and controls over growth of other bacteria [45].

The results obtained here are in contradiction with studies by Sexton [51], Ebersole [52], Yousefimanesh [53], Gursoy [54], and Reis [55]. They reported TNF-α concentration was not affected by non-surgical therapy. In Sexton and Ebersole studies, being a smoker was not among exclusion criteria. But, cigarette smoking can affect TNF-α level in gingival sulcus and saliva.

The controversies seen between studies can be due to several factors including difference in type and severity of periodontal disease, age of the subjects, salivary sample or gingival sulcus sample, the temperature at which the samples were stores, storage time before laboratory assay, and centrifuge setting. In some studies (Ebersole and Sexton studies), Multiplex ELISA method was applied.

The current results showed that salivary IL-1α level was statistically different between periodontitis and control group. In fact, salivary level of this factor was higher in control group. With scaling, salivary IL-1α increased and approached to the control group, but this was not statistically different.

Shy study reported that salivary IL-1α level increased in patients who responded to treatment [30]. In Front study, IL-1α level showed a decrease by 51% after treatment. In this study, no control group was included [56]. The difference between the results of the mentioned study and our results can be due to difference in the time when the second sample was collected. In Front study, the second sample was obtained 10 to 14 weeks after therapy. The results of Reis study also showed that IL-1α level increased significantly after treatment. However, Reis showed significant decrease in IL-1α level obtained from gingival sulcus [55]. This difference in the kind of mouth fluid samples can be another factor justifying the difference observed between studies. Saliva is a combination of secretions from the salivary glands, the gingival sulcus fluid, and oral cavity cells. Studying pro-inflammatory factors concentration in salivary samples exhibit results which are in contrast to common belief about the role that the immune system and inflammatory markers in progression of periodontal tissue destruction.

## CONCLUSION

According to the obtained findings, salivary TNF-α and IL-1α levels were lower in patients with chronic periodontitis in comparison to control group. There was a significant association between these two factors and pocket depth and attachment loss. So, treatment of periodontal disease increased the salivary levels of these two markers.

## SUGGESTION

Regarding the differences existing between designs of studies like the method of saliva collection or the gingival sulcus fluid, temperature and time of storage of samples until ELISA is performed, and centrifuge settings, we recommend a standard method for future studies. If standard and uniform method is done, comparisons between studies can be done. We suggest conducting studies with larger sample sizes and different follow-up times to achieve more general results.

## Figures and Tables

**Table 1:** Clinical parameters of periodontitis group before and after the therapy.

Variable	BT	AT	*P* value
PD (mm)	2.39 (±0.56)	1.71 (1.65 to 2.11)	< 0.001
CAL (mm)	2.93 (±0.92)	2.3 (1.7 to 2.7)	< 0.001
BOP (%)	45.5 (±20.8)	15 (5.3 to 25)	< 0.001
PI (%)	85 (72 to 95)	53.7 (±23.5)	< 0.001

Abbreviations: BT= before treatment; AT= after treatment, PD=pocket depths, CAL= clinical attachment loss, BOP= bleeding on probing, PD= periodontal index Wilcoxon test

**Table 2:** Comparison between salivary TNF-α and IL-1α before and after treatment and control group.

***P*-Value3**	***P*-Value2**	**Control Group**	***P*- Value1**	**After Treatment**	**Before Treatment**	
0.042**	0.002*	72.4±28.4	0.021*	58.02±15.33	51.06±17.77	TNF-α
0.07**	0.002*	97.54±66.04	0.056*	65.67±38.31	54.66±31.50	IL-1α

*P*-Value1= comparison between before and after treatment. *P*-Value2= comparison between before treatment and control group. *P*-Value3= comparison between after treatment and control group *u man whitney test **t-test

**Table 3:** Correlation coefficient between PD and CAL with TNF-α and IL-1α levels.

CAL	PD		
-0.311 0.017	-0/294 0.025	*R*-value *p*-value	TNF-α
-0.312 0.017	-0.299 0.023	*R*-value *p*-value	IL-1α

PD=pocket depths, CAL= clinical attachment loss
